# Phytochemical Insights into *Ficus sur* Extracts and Their Biological Activity

**DOI:** 10.3390/molecules27061863

**Published:** 2022-03-13

**Authors:** Elwira Sieniawska, Łukasz Świątek, Kouadio Ibrahime Sinan, Gokhan Zengin, Anastazja Boguszewska, Małgorzata Polz-Dacewicz, Nabeelah Bibi Sadeer, Ouattara Katinan Etienne, Mohamad Fawzi Mahomoodally

**Affiliations:** 1 Department of Natural Products Chemistry, Medical University of Lublin, Chodzki 1, 20-093 Lublin, Poland; 2 Department of Virology with SARS Laboratory, Medical University of Lublin, Chodzki 1, 20-093 Lublin, Poland; anastazjaboguszewska@umlub.pl (A.B.); malgorzata.polz-dacewicz@umlub.pl (M.P.-D.); 3 Department of Biology, Science Faculty, Selcuk University, Konya 42130, Turkey; sinankouadio@gmail.com (K.I.S.); gokhanzengin@selcuk.edu.tr (G.Z.); 4 Department of Health Sciences, Faculty of Medicine and Health Sciences, University of Mauritius, Reduit 230, Mauritius; nabeelah.sadeer1@umail.uom.ac.mu (N.B.S.); f.mahomoodally@uom.ac.mu (M.F.M.); 5 Laboratoire de Botanique, UFR Biosciences, Université Félix Houphouët-Boigny, Abidjan C 47, Côte d’Ivoire; katinan.etienne@gmail.com

**Keywords:** *Ficus*, natural products, fig, antioxidant, enzymes, phytochemistry, LC-MS, anticancer, natural antivirals

## Abstract

This study focused on the biological evaluation and chemical characterisation of *Ficus sur* Forssk. (*F. sur*) (Family: Moraceae). The methanolic and aqueous extracts’ phytochemical profile, antioxidant, and enzyme inhibitory properties were investigated. The aqueous stem bark extract yielded the highest phenolic content (115.51 ± 1.60 mg gallic acid equivalent/g extract), while the methanolic leaves extract possessed the highest flavonoid content (27.47 ± 0.28 mg Rutin equivalent/g extract). In total, 118 compounds were identified in the tested extracts. The methanolic stem bark extract exhibited the most potent radical scavenging potential against 2,2-diphenyl-1 picrylhydrazyl and 2,2′-azino-bis (3-ethylbenzothiazoline-6-sulfonic acid) (475.79 ± 6.83 and 804.31 ± 4.52 mg Trolox equivalent/g extract, respectively) and the highest reducing Cu^2+^ capacity (937.86 ± 14.44 mg Trolox equivalent/g extract). The methanolic stem bark extract substantially depressed tyrosinase (69.84 ± 0.35 mg kojic acid equivalent/g extract), α-amylase (0.77 ± 0.01 mmol acarbose equivalent/g extract), acetylcholinesterase and butyrylcholinesterase (2.91 ± 0.07 and 6.56 ± 0.34 mg galantamine equivalent/g extract, respectively) enzymes. *F. sur* extracts were tested for anticancer properties and antiviral activity towards human herpes virus type 1 (HHV-1). Stem bark infusion and methanolic extract showed antineoplastic activity against cervical adenocarcinoma and colon cancer cell lines, whereas leaf methanolic extract exerted moderate antiviral activity towards HHV-1. This investigation yielded important scientific data on *F. sur* which might be used to generate innovative phytopharmaceuticals.

## 1. Introduction

For millennia, humans have centred their lives on plants in an effort to maintain good health and treat common ailments. Even though the usage of plants was based simply on people’s intuitive understanding, owing to a lack of suitable techniques to show plants’ therapeutic potential, humans have accepted the use of many medicinal plants and included them in contemporary pharmacotherapy [1]. The Royal Botanic Gardens at Kew’s Bob Allkin recognised around 28,000 plant species as medicinal plants [2]. Since the eureka moment of the discovery of Taxol, the blockbuster anti-cancer medicine produced from the Pacific yew tree, plants have demonstrated their healing ability [3]. Since then, medicinal plants have quickly captured the interest of scientists, resulting in the increased screening of medicinal plants and the use of health-promoting plant extracts in nutrition.

In line with the present worldwide trend, the medicinal plant that this study sought to investigate was *Ficus sur* Forssk. (*F. sur*) (Family: Moraceae). The genus *Ficus* comprises more than 800 species and is generally distributed in tropical and sub-tropical areas [4]. Morphologically, it is a tree that can grow up to 25–30 m tall, with leafy twigs 2–5 mm thick, puberulous, hirtellous, tomentose or hirsute to glabrescent, with the periderm typically not flaking off when dry [5]. *F. sur*, with the common names Cape fig and broom cluster fig, is used to treat a variety of ailments in many countries. Its leaves and roots are used to cure leukoderma, leprosy, wounds, oedema, respiratory problems, diarrhoea, sexually transmitted illnesses, tuberculosis, anaemia, epilepsy, rickets, dysentery, male infertility, and gonorrhoea in Sudan and Nigeria [6]. According to ethnobotanical research, *F. sur* is also used to cure swellings [7]. It has long been used in South Africa and other nations to treat renal disorders and as a natural diuretic product [8,9]. In Ethiopia, pulverized fresh *F. sur* leaves combined with water were administered orally as a traditional medicine for urine retention, effectively alleviating the condition by boosting urine production. There is also a traditional belief that the root of this plant may be utilized to treat bladder diseases [10,11]. Despite the reported beneficial effects of members of the genus *Ficus*, several side effects (particularly when eating fruits) have been reported. These include stomach problems, obstructions in the intestine and liver damage [8]. It is necessary not to exceed the recommended dose for the fruits.

The findings of Ayele and co-workers are consistent with the traditional usage of *F. sur* as a diuretic agent. The crude leaf extracts enhanced urine excretion and urinary electrolyte concentrations in a dose-dependent manner [12]. The results of another study indicated that an ethanol extract of *F. sur* has a substantial anticonvulsant effect, validating the traditional use of the plant in the treatment of epilepsies; processes may entail interaction with GABAergic, glycinergic, serotonergic, and glutaminergic system components [13]. The purpose of another study was to see how feeding a mixture of varying amounts of *F. sur* fruits and ground maize grain affected intake, digestibility, growth, and blood profile in Yorkshire pigs [14]. The findings demonstrated that the health of the pigs was better when fed with *F. sur* fruits as creatinine and cholesterol concentrations were lower.

There are reports describing the antiviral potential of different species of *Ficus*. Ethanolic extracts from *F. benjamina* leaves inhibited human herpes virus type 1 (HHV-1, HSV-1) and type 2 (HHV-2, HSV-2), and varicella-zoster virus (HHV-3, VZV), while fruit extracts were active only against HHV-3 [15]. *F. carica* latex inhibited caprine herpes virus-1 (CpHV-1) replication in MDBK cells [16], as well as HHV-1, echovirus type 11 (ECV-11) and adenovirus (ADV) replication in VERO cells [17]. Leaf methanol extract from *F. septica* impeded dengue virus (DENV) replication in various infected cell types [18], and *F. religiosa* bark extracts showed activity against human rhinovirus (HRV) and human respiratory syncytial virus (RSV) [19], and HHV-2 [20]. Recently, ethanol extract of *Ficus fistulosa* leaves was reported to show anti-HIV activity [21].

Our aims with this study were to screen methanolic and aqueous leaves and stem bark extracts of *F. sur* for antioxidant and anti-enzymatic activities. Additionally, since the absence of detailed characterisation can markedly limit our understanding of their biological activities, the extracts were subjected to detailed phytochemical profiling. The search for new cholinesterase inhibitors (acetyl- and butyryl-cholinesterase) and carbohydrate digesting inhibitors (α-amylase and α-glucosidase) is now underway and our work has evaluated several extracts of *F. sur* for probable anti-cholinesterase and antidiabetic activities. Since multiple *Ficus* species were shown to possess antiviral activity, we have undertaken the attempt to evaluate the anti-HHV-1 activity of extracts obtained from *Ficus sur*. Furthermore, we have evaluated the anticancer potential of this plant species against cervical adenocarcinoma and colon cancer cell lines. We hope that the information offered here will assist in bridging a research gap and, as a result, open up new research opportunities, notably in the production of medicinal bioproducts.

## 2. Results and Discussion

### 2.1. Bioactive Compounds

The polarity of the solute of interest was considered in choosing the solvents used to extract bioactive compounds from plants because a solute with equivalent polarity to the solvent will be sufficiently dissolved according to the law of similarity and intermiscibility [22,23]. In this study, polyphenolic compounds, such as phenolic compounds and flavonoids, were quantified from the methanolic and aqueous extracts of *F. sur*. The results are presented in Table 1.

**Figure 1:**
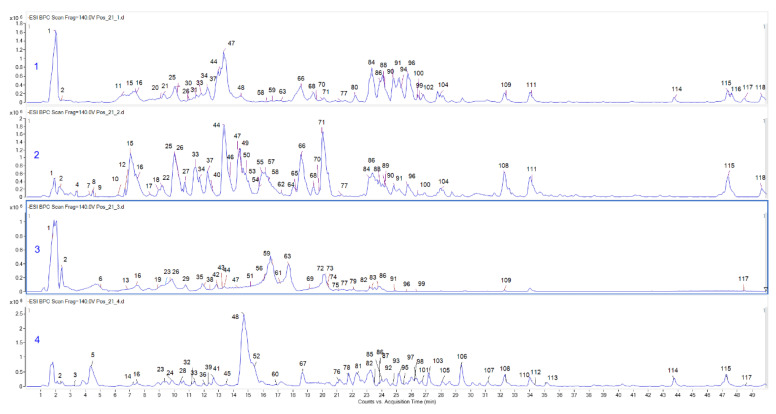
Base peak chromatograms of studied extracts. 1 *Ficus sur* leaves-MeOH; 2 *Ficus sur* leaves-infusion; 3 *Ficus sur* stem bark -MeOH; 4 *Ficus sur* stem bark -infusion.

Among the samples studied, the aqueous extract obtained from the stem bark yielded the highest amount of phenolics (115.51 ± 1.60 mg GAE/g), while the methanolic extract prepared from leaves had the highest flavonoid content (27.47 ± 0.28 mg RE/g). LC-ESI-QTOF-MS/MS analysis enabled the chemical characterization of all studied extracts. In total, 118 compounds were described (Table 2, Figure 1). It was observed that the leaf extracts contained more compounds compared to the stem bark extracts. Phenolic acids (such as quinic, citric, dihydroxybenzoic, 3-*O*-caffeoylquinic, and 5-*O*-caffeoylquinic acid), quercetin glycosides (quercetin-*O*-di-rhamnosyl-glucoside/galactoside, quercetin-*O*-glucoside, quercetin-*O*-pentoside (arabinoside), quercetin-*O*-glucuronide), and fatty acids (hydroxy octadecatrienoic acid, palmitic acid derivative) were present in almost all extracts. The methanolic extract from leaves contained mainly phenolic acids and their derivatives, esters of phenolic acids and flavonoids, and flavonoid glycosides (esters of kaempferol and quercetin). Hydroxycoumarin and methyl gallate were present only in this extract. Hydroxycaffeoylquinic, glucogallic, 2-isopropylmalic, tartaric, coumaric, ferulic acids and their esters were characteristic for leaf infusion. The methanolic extract from the stem bark was abundant in tannins, represented by catechins and procyanidins. In the stem bark infusion, apigenin, luteolin, kaempferol, quercetin and their conjugates with one or more sugar moieties dominated. The majority of the detected secondary metabolites were typical for previously studied *Ficus* species: *F. lyrata*, *F. benghalensis*, *F. benjamina*, *F. mysorensis*, *F. afzelii*, *F. pyriformis*, *F. racemose*, *F. lutea*, *F. auriculata*, *F. trigonata*, *F. spragueana*, *F. microcarpa var. nitida*; *F. virens* and *F. religiosa* for which the presence of flavonoids, flavonolignans, anthocyanins and hydroxycinnamic acids derivatives was reported [24,25].

### 2.2. Antioxidant Effects

The role of oxidative stress in the initiation and progression of human diseases supports the systemic antioxidant assessment of plant extracts under investigation. Antioxidants can perform various functions, including hydrogen atom transfer, single electron transfer, and transition metal chelation [38]. In this study, a battery of antioxidant assays was used to obtain a comprehensive understanding of the antioxidant activities of the prepared extracts of *F. sur*. The assays were: 2,2-diphenyl-1-picrylhydrazyl (DPPH), 2,2′-azino-bis (3-ethylbenzothiazoline-6-sulfonic acid) (ABTS), ferric ion reducing antioxidant power (FRAP), cupric reducing antioxidant capacity (CUPRAC), metal-chelating and total antioxidant capacity (phosphomolybdenum). As previously discussed, each test has its own set of advantages and disadvantages [38]. The results are given in Table 3.

Overall, irrespective of the type of extraction solvents used, stem bark extracts demonstrated substantially higher antioxidant activities with DPPH, ABTS, CUPRAC, FRAP, and phosphomolybdenum. For instance, methanolic stem bark extract exhibited the highest DPPH radical scavenging activities (475.79 ± 6.83 mg TE/g). The ABTS assay showed that both methanolic (804.31 ± 4.52mg TE/g) and aqueous stem bark (804.91 ± 5.45 mg TE/g) extracts demonstrated remarkably high activities. ABTS can function with lipophilic and hydrophilic molecules, but DPPH can only be solubilized in organic environments [39]. Our findings confirm the findings of Kim et al. [39]. For example, the DPPH test identified the methanolic extract as the most active, while ABTS identified both methanolic and aqueous extracts as effective ABTS scavengers.

The antioxidant capacity of the extracts was further evaluated in terms of power reduction using the CUPRAC and FRAP tests. Several variables influence antioxidants’ reducing potential, including their ionization potentials, the spin distribution of radical cations, and the bond dissociation energy of the phenolic O-H bond [40]. From Table 3, it can be seen that the methanolic stem bark extract possessed the most potent Cu^2+^ reducing potential (937.86 ± 14.44 mg TE/g) while the aqueous stem bark extract (614.33 ± 2.79 mg TE/g) was the most robust Fe^3+^ reducer.

Secondary metabolites are known to have powerful antioxidant properties due to their ability to provide electrons and because they chelate transition metals [41]. Data shown in Table 3 show that the aqueous leaves extract exhibited the highest chelating abilities (22.95 ± 0.20 mg EDTAE/g) while the methanolic stem bark extract displayed the lowest activity (4.62 ± 0.64 mg EDTAE/g). The prepared samples were also tested for their total antioxidant capacity (phosphomolybdenum assay). The latter test is based on antioxidants reducing Mo (VI) to Mo (V), resulting in the formation of a green complex in acidic conditions [42]. The aqueous stem bark extract showed the highest capacity (5.05 ± 0.05 mmol TE/g). It is noteworthy that the stem bark extracts showed stronger antioxidant ability than the leaf extracts for all assays, except the metal-chelating assay. Consequently, it can be said that the antioxidant activity of the active samples could be associated with the presence of bioactive compounds.

### 2.3. Enzymatic Inhibitory Activities

In the present study, the ability of *F. sur* extracts to modulate the activity of enzymes related to Alzheimer’s disease [acetylcholinesterase (AChE) and butyrylcholinesterase (BChE)], diabetes type 2 (α-amylase and α-glucosidase), and skin hyperpigmentation (tyrosinase) was investigated. The results are presented in Table 4.

Because enzymes in the human body contribute to the genesis of disease, inhibiting these enzymes can be advantageous in health care. Cholinesterase inhibitors, for example, are drugs that prevent the breakdown of acetylcholine, a neurotransmitter in the central nervous system that, when present in excessive concentrations, can cause neurodegenerative diseases, such as Alzheimer’s and Parkinson’s disease [43]. Our study explored the anti-cholinesterase activity in various extracts of *F. sur*. High anti-AChE and anti-BChE activities were recorded with the methanolic stem bark extract (2.91 ± 0.07 and 6.56 ± 0.34 mg GALAE/g, respectively). However, the aqueous leaves extract was inactive against AChE and BChE.

Inhibitors of α-amylase and α-glucosidase diminish carbohydrate digestion in the small intestine and, as a result, lower postprandial blood glucose levels, making them an essential therapy option for type II diabetes patients [44]. The methanolic stem bark extract of *F. sur* was observed to substantially depress α-amylase (0.77 ± 0.01 mmol ACAE/g) but was found to be inactive against α-glucosidase. Instead, the methanolic leaves extract showed high anti-glucosidase activity (3.98 ± 0.03 mmol ACAE/g).

Tyrosinase inhibitors help to protect the skin and prevent hyperpigmentation. They are strongly promoted by the pharmaceutical and cosmetics industries [45]. The methanolic stem bark extract displayed the strongest anti-tyrosinase activity (69.84 ± 0.35 mg KAE/g), while the aqueous leaves extract showed the lowest activity (0.35 ± 0.08 mg KAE/g). It is noteworthy that the methanolic stem bark extract showed the highest activity against four enzymes, namely AChE, BChE, tyrosinase, and α-amylase, although, the extract did not show the highest TFC and TPC.

### 2.4. Cytotoxicity Evaluation

**Figure 2:**
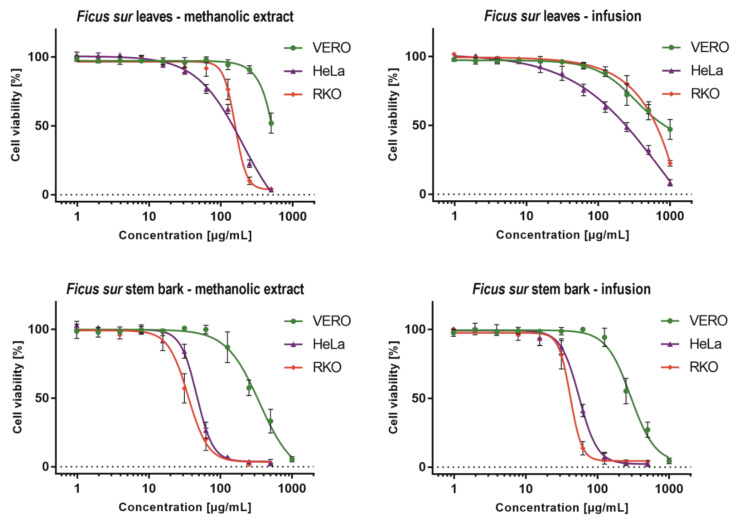
Influence of *Ficus sur* extracts on normal and cancer cells.

Cytotoxicity evaluation revealed that the infusion and methanolic extract from *Ficus sur* leaves exerted low toxicity on normal kidney fibroblasts (VERO); the exact CC_50_ values could not be evaluated because they were above the tested concentration range (Table 5). Stem bark extracts showed a similar effect on VERO cells. Selective toxicity towards HeLa cancer cells was observed for *Ficus sur* leaves methanolic extract (FLM) and infusion (FLI) with SI of >3.62 and >2.36. In contrast, in the case of RKO, only FLM showed selective toxicity (SI > 3.13). Significant antineoplastic activity towards both cancer cell lines was observed (Figure 2) for *Ficus sur* stem bark methanolic extract (FSBM) and infusion (FSBI) with CC_50_ values ranging from 36.8 to 56.12 µg/mL. The anticancer selectivity of FSBM and FSBI towards HeLa cells was 7.1 and 9.24, respectively, whereas against RKO, it was found to be 5.37 and 7.01, respectively. Multiple studies describe the anticancer potential of *Ficus* spp, ex. *Ficus carica* [46,47], *Ficus salicifolia* [46], *Ficus religiosa* [48], *Ficus beecheyana* [49], *Ficus pandurata* H [50] and *Ficus exasperata* (Vahl) [51], against various cancer cell lines, however, to the best of our knowledge, this is the first report showing *Ficus sur* stem bark extracts as a possible source of antineoplastic molecules.

### 2.5. Antiviral Potential

**Figure 3:**
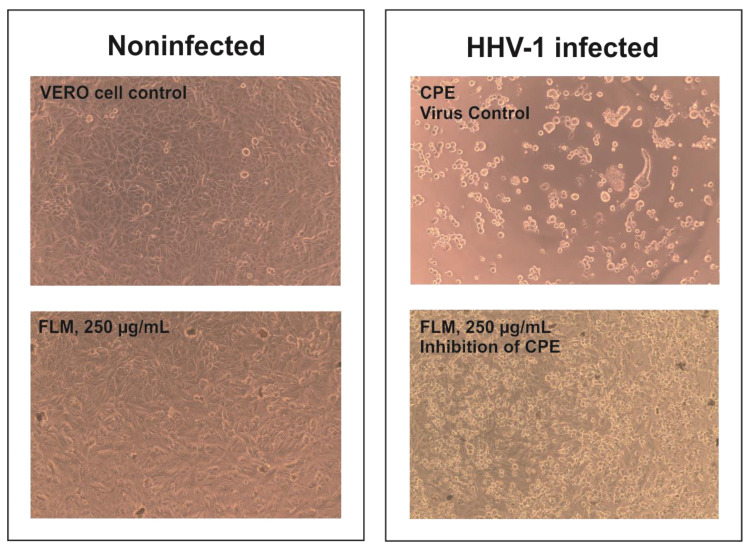
Influence of *Ficus sur* leaves methanolic extract on HHV-1 generated cytopathic effect.

**Figure 4:**
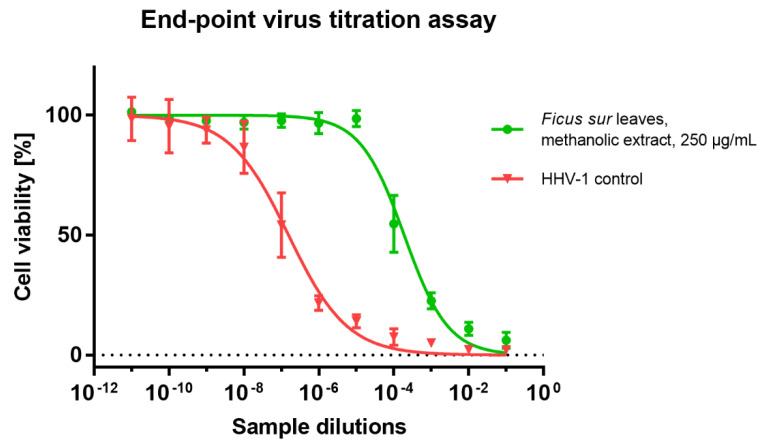
Titration assay of HHV-1 in the sample treated with *Ficus sur* leaves methanolic extract.

The *Ficus sur* extracts were incubated with an HHV-1 infected VERO cell line to evaluate the antiviral potential. After CPE was found in the virus control cells, the influence on CPE was observed in extract-treated infected cells. It was found that only one extract, namely FLM at 250 µg/mL, decreased, but did not abolish altogether, CPE formation, as can be seen in Figure 3. The collected samples were further subjected to an end-point dilution assay to evaluate the infectious titer of HHV-1. The data on HHV-1 titer reduction contained in Table 6 confirmed that FLM 250 µg/mL exerted antiviral activity, decreasing the infectious titer by 2.86 log. However, since it is generally agreed that the tested sample should reduce the infectious titer by at least 3 log to show significant antiviral potential, FLM cannot be regarded as such. However, plant extracts are complex mixtures of compounds belonging to various groups of secondary metabolites, and the biological activity of such extracts depends on their composition, and the relative amount of particular substances and possible biological interactions (ex. synergism or antagonism). One of the end-point dilution assays performed for virus-infected cells treated is presented in Figure 4; in this particular experiment, the reduction of HHV-1 titer was 3.1 log. Considering this, the reported results can be regarded as interesting, and the observed antiviral activity will be further evaluated to elucidate the compounds responsible.

We have previously reported that *Oenanthe aquatica* and *Oenanthe silaifolia* extracts possess significant antiviral activity, and the observed effect may be related to the presence of caffeic acid and its derivatives (caffeic acid glucoside, chlorogenic acid, cryptochlorogenic acid, and neochlorogenic acid) present in those extracts [52]. Interestingly, caffeic acid derivatives were identified in the FLM, which showed the highest anti-HHV-1 activity, and in FSBI, which exerted a noticeable, though much lower, influence on the tested herpes virus, reducing the infectious titer only by 0.92 log. Furthermore, methyl gallate, detected exclusively in the FLM, was proven to be a potent and specific inhibitor of HHV-2 [53]. Additionally, FLM was the only extract showing the presence of 5,8-dihydroxy-7-methoxyflavone-*O*-glucoside-rhamnoside; there are reports of antiviral activity of some flavone compounds ex. 5,7-dihydroxy-3,4′-dimethoxyflavone (ermanin) and 5,7,4′-trihydroxy-3-methoxyflavone (isokaempferide) against polio [54] or 5,7,4′-trihydroxy-8-methoxyflavone against influenza virus [55], while 5-hydroxy-7-methoxyflavone and 5,7-dimethoxyflavone were found to be protease inhibitors active against HIV-1, HCV, and HCMV (Human cytomegalovirus, HHV-5, CMV) at micromolar concentrations [56]. The kaempferol-*O*-glucoside present in FLM was also isolated from *Securigera securidaca* and reported to inhibit HHV-1 attachment to the cell membrane, virus entry and viral polymerase [57], and showed potent anti-HIV-1 reverse transcriptase activity [58]. Flavone glycosides, namely quercetin-3-*O*-rutinoside, kaempferol-3-O-rutinoside and kaempferol-3-*O*-robinobioside, were reported by Yarmolinsky et al. [59] as being responsible for the antiviral potential of *Ficus benjamina*. Interestingly, isolated glycosides exerted significant antiviral activity against HHV-1 and HHV-2, especially when added to infected cells during and after infection, but no activity was found against HHV-3 (varicella-zoster virus, VZV). Flavone aglycones, kaempferol and quercetin, obtained as standards, showed significantly lower activity [59]. Finally, FLM was the only extract that showed the presence of (epi)-afzelechin-7-*O*-glucoside, and of note, ent-epi-afzelechin-(4-8)-epiafzelechin was reported to inhibit HHV-2 by disrupting virus penetration and interfering with late stages of the viral replication cycle [60].

## 3. Materials and Methods

### 3.1. Plant Materials and Preparation of Extracts

*Ficus sur* samples were collected in the village of Prikro (city of Brobo, Côte d’Ivoire), in January 2020. The species was identified by a plant taxonomist at the National Floristic Center (Universite Felix Houphouet Boigny, Abidjan, Côte d’Ivoire). Voucher specimens were deposited at the herbarium of the above-mentioned center. The leaves and stem barks of the plant samples were dried in shade conditions at room temperature for about one week. Then, the samples were powdered with a mill and stored in dark conditions.

Different solvents (methanol and water) were used to obtain the extracts in this study. Maceration was used as the extraction method for methanol extracts. In addition, the infusion was prepared. For the maceration, the plant materials (10 g) were macerated with 200 mL methanol at room temperature overnight. After that, the mixtures were filtered, and the solvents were evaporated. In preparing the water extracts, the plant materials (10 g) were kept with 200 mL boiled water for 15 min and then filtered. Water extracts were lyophilized, and all extracts were stored at 4 °C until analysis.

### 3.2. Chromatographic Conditions

The separation was performed on a C18 Gemini^®^ column (3 µm i.d. with TMS end-capping, 110 Å, 100 × 2 mm) supported with a guard column (Phenomenex Inc, Torrance, CA, USA), at a flow rate of 0.2 mL/min under a gradient program operated by Agilent 1200 Infinity HPLC (Agilent Technologies, Santa Clara, CA, USA). Solvent A was water with 0.1% formic acid (*v*/*v*), whereas solvent B was 0.1% formic acid in acetonitrile (*v*/*v*). Both solvents were mixed according to the following program: 0–60% B for 45 min., next 60–95% B for 1 min., and 95% B for 4 min. The stop time was at 50 min. 10 μL of the sample was injected into a thermostated (20 °C) chromatographic column.

### 3.3. Detection Conditions

Mass spectra were acquired by the Agilent 6530B QTOF Accurate-Mass QTOF system equipped with Dual Agilent Jet Stream spray source (ESI) (Agilent Technologies, Santa Clara, CA, USA) connected with N_2_ generator (Parker Hannifin Corporation, Haverhill, MA; generating N_2_ at purities >99%). Negative ion mode was applied for MS and MS/MS acquisition with drying gas temp: 275 °C, drying gas flow: 10 L/min, sheath gas temp: 325 °C, sheath gas flow: 12 L/min; nebulizer pressure: 35 psig, capillary V (+): 4000 V, skimmer 65 V, fragmentor 140 V. Two spectra per sec were recorded in a range between 100 and 1000 *m*/*z* with a collision energy of 10 and 40 eV. The identification of compounds was based on fragmentation patterns and supported by a comparison of obtained mass spectra with those available in databases and the scientific literature.

### 3.4. Total Phenolic and Flavonoid Content

Total levels of phenolics and flavonoids were assessed based on previously reported methods [61,62]. Total phenolic levels were expressed as mg gallic acid equivalents (GAE)/g dry extract, and mg rutin equivalents (RE)/g dry extract was used to evaluate total flavonoids. All experimental details are given in the Supplementary Materials. The experiments were performed in triplicate, and the results were assessed by ANOVA assays (Tukey’s test).

### 3.5. Antioxidant and Enzyme Inhibitory Assays

In the current investigation, the antioxidant effects of the tested extracts were detected by different assays [61]. The assays were: [1,1-diphenyl-2-picrylhydrazyl (DPPH) and 2,2′-azino-bis(3-ethylbenzothiazoline) 6-sulfonic acid (ABTS) radical scavenging, cupric ion reducing antioxidant capacity (CUPRAC), ferric ion reducing antioxidant power (FRAP), metal chelating ability (MCA) and phosphomolybdenum assay (PDA)]. For DPPH, ABTS, CUPRAC and FRAP assays, data were expressed as mg Trolox equivalents (TE)/g extract, whereas in MCA and PDA, mg EDTA equivalents (EDTAE)/g extract and mmol TE/g extract, respectively, were used. The experimental details for acetylcholinesterase, butyrylcholinesterase, tyrosinase, amylase and glucosidase assays were previously provided. Galanthamine was used as a positive control in cholinesterase assays, and data were evaluated as mg galanthamine equivalents (GALAE)/g extract. Kojic acid was used as a standard inhibitor in tyrosinase inhibitory assay, and the results were expressed as mg kojic acid equivalents (KAE)/g extract [61,62]. Acarbose was selected as an inhibitor of both amylase and glucosidase in the antidiabetic assays, and the results are given as mmol acarbose equivalents (ACAE)/g extract. All experimental details are given in the Supplementary Materials. The assays were performed in triplicates, and the differences in the extracts were evaluated by ANOVA (Tukey’s test).

### 3.6. Cytotoxicity Testing

The evaluation of cytotoxicity was performed against normal kidney fibroblasts (VERO) and cancer cell lines derived from cervical adenocarcinoma (HeLa) and colon cancer (RKO) using microculture tetrazolium assay (MTT) as previously described [52]. Briefly, the cell monolayers were incubated with serial dilutions of the tested extracts for 72 h, and then cellular viability was assessed using the MTT protocol. Details can be found in the Supplementary Materials. The collected data were analyzed using GraphPad Prism to calculate the CC_50_ values (50% cytotoxic concentration). Additionally, selectivity indexes (SI) were calculated by comparing CC_50_ values obtained for VERO with those observed for cancer cells (SI = CC_50_VERO/CC_50_Cancer, SI > 1 indicates selectivity towards cancer cells).

### 3.7. Evaluation of Antiviral Potential

The extracts in non-toxic concentrations were tested for their influence on HHV-1 replication in the virus-infected VERO cells after 72 h incubation as previously described [52]. Briefly, the monolayer of VERO cells was treated with HHV-1 (100-fold CCID_50_, CCID_50_–50% cell culture infections dose) for 1 h, followed by washing with PBS (phosphate-buffered saline) and further incubated until a cytopathic effect (CPE) was recorded in the virus control (VC). Subsequently, after three cycles of freezing (−72 °C) and thawing, the HHV-1 infectious titer in the collected samples was measured using an end-point titration assay. Finally, the HHV-1 titer (Δlog) difference was calculated (Δlog = logCCID_50_VC–logCCID_50_FE, FE-Ficus extract). The difference of ≥3 log is regarded as significant.

## 4. Conclusions

In conclusion, the *F. sur* methanolic stem bark extract demonstrated substantial in vitro antioxidant potential with DPPH, ABTS, and CUPRAC assays, but not with FRAP, metal-chelating and phosphomolybdenum assays. The methanolic stem bark extract significantly depressed tyrosinase, α-amylase, AChE and BChE activity. To date, no evidence of enzyme inhibitory actions of *Ficus* members has been discovered. In this regard, the presented work is the first scientific demonstration of the enzyme inhibitory effects of *F. sur* extracts, and it may offer a substantial contribution to the scientific platform. Herein, we would like to report that the *F. sur* leaves methanolic extract exerted noticeable, but limited, antiviral activity against HHV-1, diminishing CPE development and reducing the virus titer by 2.86 log. Furthermore, antineoplastic activity against cervical adenocarcinoma and colon cancer cell lines was observed for stem bark infusion and methanolic extract. However, more study, including in vivo and clinical investigations, is needed to further examine these aforementioned properties to incorporate this traditional herb as a possible therapeutic element.

## Figures and Tables

**Table 1:** Total phenolic and flavonoid contents of *Ficus sur* extracts.

Parts	Solvents	TPC (mg GAE/g)	TFC (mg RE/g)
Leaves	MeOH	58.46 ± 0.28 ^c^	27.47 ± 0.28 ^a^
	Infusion	51.77 ± 0.77 ^d^	16.65 ± 0.18 ^b^
Stem barks	MeOH	109.79 ± 2.19 ^b^	2.54 ± 0.10 ^c^
	Infusion	115.51 ± 1.60 ^a^	1.13 ± 0.11 ^d^

Values are reported as mean ± SD of three parallel measurements. GAE: Gallic acid equivalents; RE: Rutin equivalents. Different letters in the same column indicate significant differences in the teste extracts (*p* < 0.05).

**Table 2:** Compounds identified in the studied extracts.

Comp. No	Tentative Identification	R Time	Molecular Mass	[M − H]^−^	Fragment Ions (*m*/*z*)	Extracts
1	Quinic acid	1.91	192.0507	191.0507	173.0464; 111.0437; 93.0318; 85.0262	1,2,3
2	Citric acid	2.33	192.0180	191.0180	111.0035; 87.0052	1,2,3,4
3	Caffeic acid derivative	3.40	242.0302	241.0302	179.0273; 153.0497; 135.0406; 123.0407; 109.0230	4
4	Quinic acid derivative	3.46	534.1621	533.1621	337.0845; 191.0508	2
5	Quinic acid derivative	4.04	406.0968	405.0968	213.0351; 191.0511	4
6	4-Hydroxy-2-(hydroxymethyl)benzoic acid	4.09	168.0307	167.0307	149.0234; 123.0429	3
7	Dihydroxybenzoic acid glucoside derivative	4.13	532.0929	531.0929	353.0781; 315.0642; 153.0155; 96.9570	2
8	Quinic acid derivative	4.22	470.0574	469.0574	435.1422; 371.0962; 191.0423	2
9	Hydroxycaffeoyl-quinic acid	4.76	372.0900	371.0900	353.0840; 197.0360; 191.0533; 179.0312; 173.0431;135.0409	2
10	Glucogallic acid/Glucosyl gallate	6.32	332.0607	331.0607	169.0124; 151.0003; 125.0211	2
11	Dihydroxybenzoic acid glucoside derivative	6.49	436.0075	435.0075	315.0710; 153.0056	1
12	Dihydro-caffeoyl-quinic acid	6.56	356.0959	355.0959	191.0522; 181.0167; 173.0451; 137.0164; 111.0044	2
13	Hydroxybenzoic acid derivative	6.77	432.1224	431.1124	137.0243; 93.0383	3
14	Caffeoyl-hydroxybenzoic acid	7.23	300.0717	299.07117	239.0562; 179.0356; 137.0228	4
15	Dihydroxybenzoic acid glucoside isomer 1	7.487	316.0628	315.0628	153.0134; 152.0105; 108.0213	1,2
16	Dihydroxybenzoic acid	7.59	154.0143	153.0143	109.0302; 108.0225	1,2,3,4
17	Coumaric acid-hexoside-pentoside	8.48	458.0892	457.0892	325.0865; 163.0347	2
18	Dihydroxybenzoic acid glucoside isomer 2	8.51	316.0660	315.0660	153.0147; 109.0258	2
19	3-Hydroxy-4-methoxymandelate glucoside	8.86	360.0924	359.0924	197.0449; 182.0215; 153.0557; 138.0321; 123.0129	3
20	Caffeic acid derivative hexoside	9.06	376.0601	375.0601;	341.1069; 213.0650; 201.0144; 179.0316; 135.0409	1
21	Quinic acid derivative	9.08	372.0868	371.0868	251.0544; 191.052; 167.0327	1
22	2-Isopropylmalic acid	9.19	176.0571	175.0571	157.0486; 115.0371; 85.0638	2
23	Hydroxybenzoic acid	9.29	138.0208	137.0208	108.0231	3, 4
24	Unidentified	9.39	376.1236	375.1236	312.0725; 169.0827; 151.0726	4
25	Hydroxybenzoic acid 4-*O*-glucoside	10.17	300.0669	299.0669	137.0203; 93.0315	1,2
26	3-*O*-Caffeoylquinic acid	10.36	354.0782	353.0782	191.0578; 179.0370; 161.0243; 135.0461	1,2,3
27	Dihydroxybenzoic acid glucoside isomer 3	10.69	316.0660	315.0660	153.0144; 109.0258	2
28	Caffeic acid glucoside	10.76	342.0825	341.0825	179.0357; 161.0246; 135.0457	4
29	Dihydroxybenzoic acid *O*-glucoside-pentoside	10.84	448.1382	447.1382	315.1063; 153.0548; 109.0301; 108.0249	3
30	Hydroxycoumarin	10.93	340.0715	339.0715	177.0178	1
31	Methyl gallate	11.02	184.0234	183.0234	168.0071; 124.0155; 78.0128	1
32	Hydroxybenzoic acid	11.13	138.0210	137.0210	109.0209; 108.0308; 93.0345	4
33	Glucogallic acid/Glucosyl gallate	11.50	332.0607	331.0607	169.0113;168.0047;125.0225	1,2,4
34	3-*O*-*p*-Coumaroylquinic acid	11.30	338.0840	337.0840	191.0540; 163.0384	1,2
35	Procyanidin B (dimer of (epi)catechin)	11.62	578.1270	577.1270	451.1080; 425.0892; 407.0834; 289.0734; 245.0811; 125.0239	3
36	Caffeic and coumaric acid derivative	12.20	542.1494	541.1494	523.1466; 475.1519; 361.0900; 235.0490; 215.0901; 179.0358; 163.0398; 137.0249	4
37	1-*O*-Coumaroylquinic acid	12.28	338.0840	337.0840	191.0509; 173.0417; 163.0356	1,2
38	Glucogallic acid tartaric ester	12.33	464.1040	463.1040	331.0608; 169.0147; 168.0037; 149.9937; 125.0212	3
39	Caffeic acid glucoside	12.34	342.0825	341.0825	179.0344; 161.0230; 135.0440	4
40	Gallic acid glucoside derivative	12.55	412.0173	411.0173	367.355; 331.0632; 240.9965; 169.0091; 125.0157	2
41	Caffeic acid derivative hexoside	12.63	542.1494	541.1494	379.0983; 179.0323; 161.0220; 135.0425	4
42	Methyl gallate hexoside-pentoside	12.70	448.1542	447.1542	345.1151; 183.0660; 168.0382; 161.0437	3
43	(Epi)gallocatechin	13.18	306.0612	305.0612	287.0562; 269.0489; 219.0678; 195.0310; 179.0321; 161.0246; 137.0237; 125. 0235	3
44	5-*O*-Caffeoylquinic acid	13.27	354.0811	353.0811	191.0521; 161.0214; 135.0406	1,2,3
45	Dihydroxycoumarin	13.47	178.0152	177.0152	161.0960; 133.0279; 105.0338	4
46	3-*O*-Feruloylquinic acid	13.66	368.0964	367.0964	193.0481; 173.0430; 134.0355	2
47	4-*O*-Caffeoylquinic acid	14.32	354.0811	353.0811	191.0516; 179.0306; 173.0412; 135.0414	2
48	Caffeic acid	14.41	180.0293	179.0293	135.0451	1,4
49	Coumaroyltartaric acid isomer 1	14.68	296.0404	295.0404	163.0357; 149.0051; 130.9957; 119.0460	2
50	Tartaric acid	14.94	150.0055	149.0055	130.9997; 102.9993; 87.0065	2
51	4-*O*-Methylgallocatechin	15.25	320.0763	319.0763	287.0497; 243.0251; 197.0451; 161.0242; 125.0233	3
52	Caffeic and hydroxycinnamic acid derivative	15.39	380.0995	379.0995	369.0971; 251.0590; 217.0658; 179.0345; 161.0261; 135.0426	4
53	Coumaric acid hexoside derivative	15.49	442.0969	441.0969	325.0912; 163.0378; 119.0507	2
54	Coumaric acid	15.76	164.0361	163.0361	119.0483	2
55	Coumaroyltartaric acid isomer 2	15.82	296.0404	295.0404	163.0363; 149.0056; 130.9944; 119.0472	2
56	B-type procyanidin trimer	16.03	866.1873	865.1873	739.1731; 713.1525; 577.1396; 425.0996; 287.0524; 245.0479; 125.0216	3
57	Caffeic acid pentoside	16.15	312.0340	311.0340	179.0036; 135.0380	2
58	4-*O*-*p*-Coumaroylquinic acid	16.33	338.0871	337.0871	191.0520; 173.0423; 163.0363	1,2
59	Procyanidin B (dimer of (epi)catechin)	16.54	578.1226	577.1226	451.0947; 425.0842; 407.0791; 289.0774; 245.0500	1,3
60	Caffeic acid and hydroxycoumarin derivative	16.93	458.1282	457.1282	383.0989; 221.0481; 179.0362; 161.0255; 135.0433; 133.0275; 117.0335	4
61	(Epi)catechin derivative	17.11	326.0425	325.0425	289.0667; 245.0790; 205.0449; 125.0275	3
62	Sinapoyl-ferulate	17.22	400.0876	399.0876	223.0469; 205.0374; 193.0429; 129.0150; 111.0050; 85.0271	2
63	(Epi)catechin	17.26	290.0637	289.0637	245.0785; 205.0476; 187.0356; 179.0295; 125.0258	1,3
64	Ferulic acid pentoside	18.03	326.0500	325.0500	193.0474; 178.0238; 134.0347	2
65	Caffeoylshikimic acid	18.03	336.0713	335.0713	179.0325; 173. 0429; 161.0208; 135.0420	2
66	5-*O*-*p*-Coumaroylquinic acid	18.50	338.0871	337.0871	191.0514; 173.0417; 163.0356	1,2
67	Unidentified	18.67	578.1268	577.1268	541.1482; 379.0918; 179.0298; 161.0208; 135.0406	4
68	Caffeoylshikimic acid isomer	19.17	336.0713	335.0713	179.0302; 173. 0411; 161.0200; 155.0291; 135.0415	1, 2
69	B-type procyanidin trimer	19.20	866.1873	865.1873	739.1553; 713.1344; 577.1240; 425.0756; 287.0531; 245.0383; 125.0183	3
70	4-*O*-Feruloylquinic acid	19.75	368.0972	367.0972	191.0531; 173.0423	1,2
71	Caffeoylmalic acid	20.07	296.0416	295.0416	179.0315; 133.115; 115.0010	1,2
72	Procyanidin B (dimer of (epi)catechin) derivative	20.07	880.1656	879.1656	727.1275; 577.1073; 439.0638; 407.0538; 287.0468; 245.0405; 125.0207	3
73	B-type procyanidin trimer	20.38	866.1873	865.1873	739.1553; 713.1344; 577.1292; 451.0938; 425.0813; 407.0716; 287.0522; 245.0383; 125.0207	3
74	(Epi)catechin-(epi)gallocatechin	20.55	594.1023	593.1223	575.1061; 467.1034; 441.0792; 423.0659; 305.0549; 287.0417; 245.0385; 125.0204	3
75	Procyanidin B (dimer of (epi)catechin) derivative	20.76	721.6486	720.6486	644.1217; 577.1295; 289.0645; 245.0399; 125.0202	3
76	Quercetin-*O*-di-glucoside	21.27	626.1342	625.1342	463.0867; 301.0301; 300.0238; 178.9961; 151.0005	4
77	Quercetin-*O*-di-rhamnosyl-glucoside/galcactoside	21.28	756.1880	755.1880	609.1432; 300.0275; 271.0254; 178.9947; 151.0029	1,2,3
78	Unidentified	21.74	536.1387	535.1387	491.1498; 323.0715; 281.0600; 179.0314; 161.0215	4
79	Procyanidin dimer monoglycoside	22.18	740.1601	739.1601	587.1146; 459.0606; 449.0773; 435.0625; 289.062; 245.0766; 125.0214	3
80	5,8-Dihydroxy-7-methoxyflavone-*O*-glucoside-rhamnoside	22.22	592.1811	591.1811;	445.1129 325.0735; 297.0404; 293.0638; 282.0504	1
81	Quercetin-3-*O*-arabino-glucoside	22.24	596.1230	595.1230	301.0279; 300.0229; 271.0219; 255.0251; 178.9902	4
82	Rutin	23.06	610.1377	609.1377	301.0285; 300.0216; 271.0215; 178.9902; 150.9959	3,4
83	Procyanidin B (dimer of (epi)catechin)	23.25	578.1270	577.1270	451.0997; 425.0849; 407.0722; 289.0671; 125.0228	3
84	Rutin-*O*-(*p*-coumaroyl) malate	23.32	890.1862	889.1862	609.1328; 300.0192; 271.0182; 178.9951; 163.0345; 150.9965; 133.0093	1, 2
85	Quercetin derivative hexoside	23.85	566.1131	565.1131	403.1489; 301.0262; 300.0204; 271.0208; 255.0250; 178.9927; 150.9985	4
86	Quercetin-*O*-glucoside	23.86	464.0778	463.0778	300.0028; 271.0183; 178.9940; 150.9981	1,2,3,4
87	Kaempferol-*O*-pentoside-hexoside	24.08	580.1286	579.1286	447.0887; 285.0334; 284.0273; 255.0245; 227.0320; 150.9995; 133.0083	4
88	Kaempferol-3-*O*-rhamnosyl galactoside	24.21	594.1381	593.1381	285.0311; 257.0382; 255.0197; 229.0456; 187.0403	1, 2
89	Ferulic acid	24.26	194.0463	193.0463	178.0225; 149.0556; 134.0330; 117.0299	2
90	Quercetin-*O*-(glucosyl-feruloylmalate)	24.36	774.1473	773.1473	463.0811; 309.0561; 193.0466; 134.0333	1,2
91	Quercetin-*O*-pentoside (arabinoside)	24.75	434.0683	433.0686	301.0267; 300.0213; 271.0193; 255.0248; 227.0281; 150.9994	1,2,3
92	Kaempferol-C-di-hexoside-*O*-hexoside	24.85	772.1694	771.1694	609.1382; 485.1224; 429.0806; 383.0925; 323.0716; 285.0338; 255.0239; 227.0213; 161.0214; 133.0241	4
93	Luteolin-*O*-glucuronide	25.13	462.0664	461.0664	285.0343; 175.0245; 133.0246;	4
94	Kaempferol-*O*-glucoside	25.18	448.0832	447.0832	284.0261; 255.0237; 227.0277; 150.9974	1
95	Quercetin-*O*-(caffeoyl-di-glucoside)	25.44	788.1650	787.1650	625.1372; 461.0677; 387.1494; 301.0286; 300.0232; 179.0375; 161.0161	4
96	Quercetin-*O*-glucuronide	25.71	478.0832	477.0832	301.0285; 271.0556; 178.9914; 150.9982	1,2,3
97	Quercetin-*O*-arabinoside-di-glucoside	25.92	758.1530	757.1530	595.1207; 463.0830; 301.0292; 300.0215; 178.9928; 150.9968	4
98	Quercetin-*O*-glucoside-arabinoside-glucuronide	26.21	772.1694	771.1694	595.1243; 301.0275; 300.0228; 271.0201; 178.9963; 150.9999	4
99	Kaempferol-*O*-di-pentoside	26.40	550.1142	549.1142	417.0841; 285.0453	1,3
100	Kaempferol-*O*-pentoside	26.42	418.0743	417.0743	284.0376; 285.0421	1,2
101	Di-caffeoyl-dihydroxybenzoic acid	26.60	478.0983	477.0983	433.1033; 315.0667; 179.0321; 161.0204; 153.0140; 152.0098; 135.0443; 109.0295; 108.0158	4
102	(Epi)-afzelechin-7-*O*-glucoside	26.82	436.1199	435.1199	345.0936; 273.0730; 167.0336	1
103	Kaempferol-*O*-(caffeoyl-arabinoside-glucoside	27.09	742.1608	741.1608	579.1326; 455.1050; 429.0733; 285.0349; 284.0270; 255.0312; 227.0223; 179.0308; 161.0221; 135.0376	4
104	Kaempferol-*O*-rhamnoside	27.88	432.0906	431.0906	285.0431; 284.0317; 255.0287; 227.0334	1,2
105	Quercetin-*O*-glucoside-arabinoside-glucuronide	27.99	772.1694	771.1694	595.1228; 301.0258; 300.0216; 271.0204; 255.0185; 178.9945; 150.9824	4
106	Kaempferol-*O*-arabinosisde-glucoside-rhamnoside	29.28	726.1658	725.1658	579.1227; 285.0346; 284.0272; 255.0222; 227.0298	4
107	Luteolin	31.09	286.0364	285.0364	175.0368; 133.0300	4
108	Trihydroxy-octadecadienoic acid	32.36	328.2116	327.2116	291.1989; 229.1460; 211.1336; 171.1031	2,4
109	Unidentified	32.36	396.1960	395.1960	349.2045; 327.2269; 251.1307; 233.1170; 193.0888; 171.1032	1, 3
110	Trihydroxy-octadecenoic acid	33.91	330.2292	329.2292	311.2203; 293.1239; 229.1450; 211.1334; 171.1011	4
111	12-oxo-*10E*-dodecenoic acid	34.01	228.1222	227.1222	209.1179; 183.1395; 165.1298	1,2
112	Apigenin	34.28	270.0417	269.0417	227.0349; 151.0027; 117.0349; 107.0126	4
113	Trihydroxy-octadecenoic acid derivative	35.261	444.1493	443.1493	329.2280; 309.1244; 293.1891	4
114	12-oxo-*10E*-dodecenoic acid derivative	43.89	722.3519	721.3519	675.3594; 397.1348; 227.2159	1,4
115	Hydroxy octadecatrienoic acid	47.40	294.2048	293.2048	275.2015; 224.1403; 195.1388	1,2,4
116	Palmitic acid derivative	47.50	700.3671	699.3671	653.7889; 397.1369; 255.2329	1
117	Palmitic acid derivative	48.36	541.3192	540.3192	480.3059; 255.2310	1,3,4
118	Hydroxy octadecadienoic acid derivative	49.62	366.2055	365.2055	317.2080; 295.2254; 277.2110	1,2

1 *Ficus sur* leaves-MeOH; 2 *Ficus sur* leaves-infusion; 3 *Ficus sur* stem bark-MeOH; 4 *Ficus sur* stem bark -infusion. The identification was supported by the following sources [24,25,26,27,28,29,30,31,32,33,34,35,36,37].

**Table 3:** Antioxidant properties of *Ficus sur* extracts.

Parts	Solvents	DPPH (mg TE/g)	ABTS (mg TE/g)	CUPRAC (mg TE/g)	FRAP (mg TE/g)	PBD (mmol TE/g)	MCA (mg EDTAE/g)
Leaves	MeOH	48.66 ± 0.04 ^c^	81.41 ± 0.05 ^b^	160.80 ± 1.55 ^c^	108.50 ± 2.04 ^c^	1.65 ± 0.11 ^b^	7.88 ± 0.99 ^c^
	Infusion	44.22 ± 0.06 ^c^	72.32 ± 1.69 ^b^	147.58 ± 1.59 ^c^	77.28 ± 0.25 ^d^	1.65 ± 0.08 ^b^	22.95 ± 0.20 ^a^
Stem barks	MeOH	475.79 ± 6.83 ^a^	804.31 ± 4.52 ^a^	937.86 ± 14.44 ^a^	523.17 ± 2.92 ^b^	5.00 ± 0.30 ^a^	4.62 ± 0.64 ^d^
	Infusion	463.58 ± 1.17 ^b^	804.91 ± 5.45 ^a^	910.68 ± 12.14 ^b^	614.33 ± 2.79 ^a^	5.05 ± 0.05 ^a^	13.22 ± 0.18 ^b^

Values are reported as mean ± SD of three parallel measurements. TE: Trolox equivalents; EDTAE: EDTA equivalents. Different letters in the same column indicate significant differences in the tested extracts (*p* < 0.05).

**Table 4:** Enzyme inhibitory properties of *Ficus sur* extracts.

Parts	Solvents	AChE (mg GALAE/g)	BChE (mg GALAE/g)	Tyrosinase (mg KAE/g)	Amylase (mmol ACAE/g)	Glucosidase (mmol ACAE/g)
Leaves	MeOH	2.11 ± 0.24 ^b^	2.31 ± 0.21 ^b^	68.12 ± 0.47 ^b^	0.61 ± 0.01 ^c^	3.98 ± 0.03 ^a^
	Infusion	na	na	0.35 ± 0.08 ^d^	0.13 ± 0.01 ^d^	3.90 ± 0.01 ^b^
Stem barks	MeOH	2.91 ± 0.07 ^a^	6.56 ± 0.34 ^a^	69.84 ± 0.35 ^a^	0.77 ± 0.01 ^a^	na
	Infusion	1.88 ± 0.11 ^b^	na	51.55 ± 0.24 ^c^	0.74 ± 0.02 ^b^	na

Values are reported as mean ± SD of three parallel measurements. GALAE: Galantamine equivalents; KAE: Kojic acid equivalents; ACAE: Acarbose equivalents; na: not active: Different letters in the same column indicate significant differences in the tested extracts (*p* < 0.05).

**Table 5:** Results of cytotoxicity evaluation.

*Ficus sur*	Solvent		CC_50_ ± SD (µg/mL)		
			VERO	HeLa	RKO
Leaves	MeOH	FLM	>500	138.3 ± 3.78	159.97 ± 12.3
	Infusion	FLI	>500	212.0 ± 19.45	594.23 ± 41.0
Stem bark	MeOH	FSBM	340.1 ± 22.72	47.89 ± 0.27	36.8 ± 4.92
	Infusion	FSBI	301.12 ± 30.31	56.12 ± 1.89	42.96 ± 4.94

**Table 6:** Abatement of HHV-1 infectious titer in response to *Ficus sur* treatment.

*Ficus sur*	Solvent	Concentration (µg/mL)	Decrease of HHV-1 Infectious Titer (Δlog *)
Leaves	MeOH	250	2.86 ± 0.17
		125	1.03 ± 0.23
	Infusion	100	0.52 ± 0.19
		50	0.09 ± 0.16
Stem bark	MeOH	125	0.12 ± 0.06
		62.5	0.07 ± 0.25
	Infusion	125	0.92 ± 0.52
		62.5	0.35 ± 0.13

* Δlog (mean ± SD)–calculated from separate titration assays; Δlog = logCCID_50_VC–logCCID_50_E; VC–virus control; E–extract, Δlog ≥ 3 is regarded as significant.

## Data Availability

Not applicable.

## Supplementary Materials

The following supporting information can be downloaded at: https://www.mdpi.com/article/10.3390/molecules27061863/s1.
