# Supplementary material for: Phytochemical Insights into Ficus sur Extracts and Their Biological Activity
Source: Molecules. 2022 Mar 13;27(6):1863. doi: 10.3390/molecules27061863 (PMC8949149; doi:10.3390/molecules27061863)
Supplement: Supplementary file 1 [file molecules-27-01863-s001.zip › molecules-1599982-supplementary.pdf]

molecules

Supplementary Materials

Phytochemical Insights into *Ficus sur* Extracts and Their Biological Activity

Elwira Sieniawska [1,*,†], Łukasz Świątek [2,*,†], Kouadio Ibrahime Sinan [3], Gokhan Zengin [3], Anastazja Boguszewska [2], Małgorzata Polz-Dacewicz [2], Nabeelah Bibi Sadeer [4] and Ouattara Katinan Etienne [5] and Mohamad Fawzi Mahomoodally [4]

[1] Department of Natural Products Chemistry, Medical University of Lublin, Chodzki 1, 20-093 Lublin, Poland
[2] Department of Virology with SARS Laboratory, Medical University of Lublin, Chodzki 1, 20-093 Lublin, Poland; anastazjaboguszewska@umlub.pl (A.B.); malgorzata.polz-dacewicz@umlub.pl (M.P.-D.)
[3] Department of Biology, Science Faculty, Selcuk University, Konya 42130, Turkey; sinankouadio@gmail.com (K.I.S.) and gokhanzengin@selcuk.edu.tr (G.Z.)
[4] Department of Health Sciences, Faculty of Medicine and Health Sciences, University of Mauritius, Reduit 230, Mauritius; nabeelah.sadeer1@umail.uom.ac.mu (N.B.S.); f.mahomoodally@uom.ac.mu (M.F.M.)
[5] Laboratoire de Botanique, UFR Biosciences, Université Félix Houphouët-Boigny, Abidjan C 47, Côte d'Ivoire; katinan.etienne@gmail.com
* Correspondence: esieniawska@pharmacognosy.org (E.S.); ulubiony.asystent@gmail.com (Ł.Ś.)
† These authors contributed equally to this work.

Cell Line Maintenance and In Vitro Experiments

The cytotoxicity of *Ficus sur* extracts was evaluated in vitro towards normal VERO (ATCC, Cat. No. CCL-81) cells and cancer-derived cell lines—HeLa (ECACC, Cat. No. 93021013, cervical adenocarcinoma) and RKO (ATCC, Cat. No. CRL-2577, colon carcinoma), using 3-(4,5-dimethylthiazol-2-yl)-2,5-diphenyltetrazolium bromide (MTT) based protocol.

Media used for in vitro culturing included Dulbecco Modified Eagle Medium (DMEM, Corning, Tewksbury, MA, USA) used for VERO cells and Modified Eagle Medium (MEM, Corning) used for other cell lines. Cell media used in the experiments were supplemented with antibiotics (Penicillin-Streptomycin Solution, Corning) and fetal bovine serum (FBS, Corning)—10% (cell passaging) and 2% (cell maintenance and experiments). Phosphate buffered saline (PBS), and trypsin were bought from Corning, whereas MTT (3-(4,5-dimethylthiazol-2-yl)-2,5-diphenyltetrazolium bromide) and DMSO (dimethyl sulfoxide) from Sigma (Sigma-Aldrich, St. Louis, MO, USA). Incubation was conducted in a 5% CO_2 atmosphere at 37°C (CO_2 incubator, Panasonic Healthcare Co., Tokyo, Japan).

The Cytotoxicity Testing

Cytotoxicity was tested using MTT based protocol following a previously described protocol [52]. Briefly, the cells were passaged and transferred into 96-well plates (Falcon, TC-treated, Corning) and, after overnight incubation, treated with serial dilutions of extract stock solutions for 72 h. Afterwards, the media was removed, cells were washed with PBS, and 10% of MTT solution (5 mg/mL) in cell media was added, and the incubation continued for the next 4 h. Subsequently, the SDS/DMF/PBS (14% SDS, 36% DMF, 50% PBS) solvent was used (100 µL per well) to dissolve the precipitated formasane crystals, and the plates were left at 37 °C overnight. Finally, the Synergy H1 Multi-Mode Microplate Reader (BioTek Instruments, Inc. Winooski, VT, USA) with Gen5 software (ver. 3.09.07; BioTek Instruments, Inc.) was used to measure the absorbance (540 and 620 nm).

Evaluation of Antiviral Potential

The antiviral activity of *Ficus sur* extracts was tested against HHV-1 (ATCC, Cat. No. VR-260) propagated in the VERO cell line. The antiviral assays involved the influence of extracts on the formation of virus (HHV-1) induced cytopathic effect (CPE) and the evaluation of the reduction of infectious titer using the end-point virus titration.

The infectious titer of HHV-1 used in this study was 5.5 ± 0.25 $logCCID_{50}$/mL ($CCID_{50}$ − 50% cell culture infectious dose). Briefly, the VERO cells (monolayer) in 48-well plates (Falcon, clear flat bottom TC-treated, Corning) was treated (500 μL/well) with HSV-1 suspension (100* $CCID_{50}$/mL) in cell media and incubated for 1 h, leaving at least 2 uninfected wells as VERO cell control. Afterwards, the media were removed, monolayers washed with PBS, and the non-toxic concentrations of extracts, highest concentration not exceeding the CC_{10} values, diluted in cell media were added. The non-infected VERO cells (cell control) and non-treated infected cells (virus control) wells were maintained in media containing 2% FBS. The incubation was conducted until cytopathic effect (CPE) was observed (inverted microscope CKX41, Olympus Corporation, Tokyo, Japan) in virus control, usually approx. 72 h. Afterwards, the plates were observed for possible inhibition of CPE by tested extracts compared to the CPE in virus control, and the results were recorded. Lastly, the plates were thrice frozen (−72 °C) and thawed, and the samples were collected and stored at −72 °C until used in end-point virus titration assay. The antiviral properties of extracts were tested in three independent experiments.

End-Point Dilution Assay for HHV-1 Titration

Samples collected from antiviral assays were subjected to an end-point dilution assay to evaluate the HHV-1 titers. Briefly, the VERO cells (monolayer) in 96-well plates were incubated with ten-fold dilutions of samples (3 replicates) in cell media for 72 h. The daily observation was conducted to monitor the development of CPE. After the incubation, all media were removed, and the HSV-1 infectious titer for each sample was measured using the previously described MTT method. Subsequently, the difference (Δlog) of HHV-1 infectious titer ($logCCID_{50}$/mL) in the samples treated with tested *Ficus sur* extracts (FE) and in the virus control (VC) from the same experiment (Δlog = $logCCID_{50}VC$ − $logCCID_{50}FE$) were calculated. The Δlog values were evaluated for every antiviral assay, and the results were expressed further as means of viral titer reduction. A significant antiviral activity can be reported for extracts decreasing the infectious titer by at least 3 log compared to virus control [52]

Assays for Total Phenolic and Flavonoid Contents

The total phenolic content was determined by employing the methods given in the literature with some modification. Sample solution (0.25 mL) was mixed with diluted Folin-Ciocalteu reagent (1 mL, 1:9, *v*/*v*) and shaken vigorously. After 3 min, Na_2CO_3 solution (0.75 mL, 1%) was added and the sample absorbance was read at 760 nm after a 2 h incubation at room temperature. The total phenolic content was expressed as milligrams of gallic acid equivalents (mg GAE/g extract)

The total flavonoid content was determined using the $AlCl_3$ method. Briefly, sample solution (1 mL) was mixed with the same volume of aluminum trichloride (2%) in methanol. Similarly, a blank was prepared by adding sample solution (1 mL) to methanol (1 mL) without $AlCl_3$. The sample and blank absorbances were read at 415 nm after a 10 min incubation at room temperature. The absorbance of the blank was subtracted from that of the sample. Rutin was used as a reference standard and the total flavonoid content was expressed as milligrams of rutin equivalents (mg RE / g extract)

Determination of Antioxidant and Enzyme Inhibitory Effects

Antioxidant (DPPH and ABTS radical scavenging, reducing power (CUPRAC and FRAP), phosphomolybdenum and metal chelating (ferrozine method) and enzyme inhibitory activities (cholinesterase (Elmann's method), tyrosinase (dopachrome method), α-amylase (iodine/potassium iodide method), α -glucosidase (chromogenic PNPG method) and pancreatic lipase (*p*-nitrophenyl butyrate (*p*-NPB) method) were determined.

For the DPPH (1,1-diphenyl-2-picrylhydrazyl) radical scavenging assay: Sample solution (50 μL) was added to 150 μL of a 0.004% methanol solution of DPPH. The sample absorbance was read at 517 nm in a 96-well microplate after 30 min of incubation at room temperature in the dark. Trolox was used as a standard and a calibration curve was obtained by different trolox concentrations. DPPH radical scavenging activity was expressed as milligrams of trolox equivalents (mg TE/g extract).

For ABTS (2,2′-azino-bis(3-ethylbenzothiazoline) 6-sulfonic acid) radical scavenging assay: Briefly, ABTS+ was produced directly by reacting 7 mM ABTS solution with 2.45 mM potassium persulfate and allowing the mixture to stand for 12–16 min in the dark at room temperature. Prior to beginning the assay, ABTS solution was diluted in methanol to an absorbance of 0.700 ± 0.02 at 734 nm. Sample solution (25 μL) was added to ABTS solution (200 μL) and mixed. The sample absorbance was read at 734 nm in a 96-well microplate after 30 min of incubation at room temperature. Trolox was used as a standard and a calibration curve was obtained by different trolox concentrations. The ABTS radical scavenging activity was expressed as milligrams of trolox equivalents (mg TE/g extract).

For CUPRAC (cupric ion reducing activity) activity assay: Sample solution was added to premixed reaction mixture containing $CuCl_2$ (1 mL, 10 mM), neocuproine (1 mL, 7.5 mM) and NH_4Ac buffer (1 mL, 1 M, pH 7.0). Similarly, a blank was prepared by adding sample solution (25 μL) to premixed reaction mixture (200 μL) without $CuCl_2$. Then, the sample and blank absorbances were read at 450 nm in a 96-well microplate after 30 min of incubation at room temperature. The absorbance of the blank was subtracted from that of the sample. Trolox was used as a standard and a calibration curve was obtained by different trolox concentrations. CUPRAC activity was expressed as milligrams of trolox equivalents (mg TE / g extract).

For FRAP (ferric reducing antioxidant power) activity assay: Sample solution (25 μL) was added to premixed FRAP reagent (200 μL) containing acetate buffer (0.3 M, pH 3.6), 2,4,6-tris(2-pyridyl)-S-triazine (TPTZ) (10 mM) in 40 mM HCl and ferric chloride (20 mM) in a ratio of 10:1:1 (*v*/*v*/*v*). Then, the sample absorbance was read at 593 nm in a 96-well microplate after 30 min of incubation at room temperature. Trolox was used as a standard and a calibration curve was obtained by different trolox concentrations. FRAP activity was expressed as milligrams of trolox equivalents (mg TE / g extract).

For phosphomolybdenum method: Sample solution (100 μL) was combined with 3 mL of reagent solution (0.6 M sulfuric acid, 28 mM sodium phosphate and 4 mM ammonium molybdate). The sample absorbance was read at 695 nm in a 96-well microplate after 90 min of incubation at 95 °C. Trolox was used as a standard and a calibration curve was obtained by different trolox concentrations. The total antioxidant capacity was expressed as millimoles of trolox equivalents (mmol TE / g extract).

For metal chelating activity assay: Briefly, sample solution (100 μL) was added to $FeCl_2$ solution (50 μL, 2 mM). The reaction was initiated by the addition of 5 mM ferrozine (100 μL). Similarly, a blank was prepared by adding sample solution (100 μL) to $FeCl_2$ solution (50 μL, 2 mM) and water (100 μL) without ferrozine. Then, the sample and blank absorbances were read at 562 nm in a 96-well microplate after 10 min of incubation at room temperature. The absorbance of the blank was subtracted from that of the sample. EDTA (disodium edetate) was used as a standard and a calibration curve was obtained by different EDTA concentrations The metal chelating activity was expressed as milligrams of EDTA equivalents (mg EDTAE / g extract).

For Cholinesterase (ChE) inhibitory activity assay: Sample solution (100 μL) was mixed with DTNB (5,5-dithio-bis(2-nitrobenzoic) acid, Sigma, St. Louis, MO, USA) (125

µL) and AChE (acetylcholinesterase (Electric ell acetylcholinesterase, Type-VI-S, EC 3.1.1.7,Sigma)), or BChE (butyrylcholinesterase (horse serum butyrylcholinesterase, EC 3.1.1.8, Sigma)) solution (25 µL) in Tris–HCl buffer (pH 8.0) in a 96-well microplate and incubated for 15 min at 25 °C. The reaction was then initiated with the addition of acetylthiocholine iodide (ATCI, Sigma) or butyrylthiocholine chloride (BTCl, Sigma) (25 µL). Similarly, a blank was prepared by adding sample solution to all reaction reagents without enzyme (AChE or BChE) solution. The sample and blank absorbances were read at 405 nm after 10 min of incubation at 25 °C. The absorbance of the blank was subtracted from that of the sample. Galanthamine was used as a standard and a calibration curve was obtained by different galanthamine concentrations. The cholinesterase inhibitory activity was expressed as galanthamine equivalents (mg GALAE / g extract).

For Tyrosinase inhibitory activity assay: Sample solution (50 µL) was mixed with tyrosinase solution (40 µL, Sigma) and phosphate buffer (100 µL, pH 6.8) in a 96-well microplate and incubated for 15 min at 25 °C. The reaction was then initiated with the addition of L-DOPA (40 µL, Sigma). Similarly, a blank was prepared by adding sample solution to all reaction reagents without enzyme (tyrosinase) solution. The sample and blank absorbances were read at 492 nm after 10 min of incubation at 25 °C. The absorbance of the blank was subtracted from that of the sample. Kojic acid was used as a standard and a calibration curve was obtained by different kojic acid concentrations. The tyrosinase inhibitory activity was expressed as kojic acid equivalents (mg KAE/g extract).

For α-amylase inhibitory activity assay: Sample solution (50 µL) was mixed with α-amylase solution (ex-porcine pancreas, EC 3.2.1.1, Sigma) (50 µL) in phosphate buffer (pH 6.9 with 6 mM sodium chloride) in a 96-well microplate and incubated for 10 min at 37 °C. After pre-incubation, the reaction was initiated with the addition of starch solution (50 µL, 0.05%). Similarly, a blank was prepared by adding sample solution to all reaction reagents without enzyme (α-amylase) solution. The reaction mixture was incubated 10 min at 37 °C. The reaction was then stopped with the addition of HCl (25 µL, 1 M). This was followed by addition of the iodine-potassium iodide solution (100 µL). The sample and blank absorbances were read at 630 nm. The absorbance of the blank was subtracted from that of the sample. Acarbose was used as a standard and a calibration curve was obtained by different acarbose concentrations. The α-amylase inhibitory activity was expressed as acarbose equivalents (mmol ACE/g extract).

For α-glucosidase inhibitory activity assay: Sample solution (50 µL) was mixed with glutathione (50 µL), α-glucosidase solution (from *Saccharomyces cerevisiae*, EC 3.2.1.20, Sigma) (50 µL) in phosphate buffer (pH 6.8) and PNPG (4-N-trophenyl-α-D-glucopyranoside, Sigma) (50 µL) in a 96-well microplate and incubated for 15 min at 37 °C. Similarly, a blank was prepared by adding sample solution to all reaction reagents without enzyme (α-glucosidase) solution. The reaction was then stopped with the addition of sodium carbonate (50 µL, 0.2 M). The sample and blank absorbances were read at 400 nm. The absorbance of the blank was subtracted from that of the sample. Acarbose was used as a standard and a calibration curve was obtained by different acarbose concentrations. The α-glucosidase inhibitory activity was expressed as acarbose equivalents (mmol ACE/g extract).
